# Towards standardization of human adipose-derived stromal cells secretomes

**DOI:** 10.1007/s12015-023-10567-5

**Published:** 2023-06-10

**Authors:** Erika Pinheiro-Machado, Vasilena E. Getova, Martin C. Harmsen, Janette K. Burgess, Alexandra M. Smink

**Affiliations:** 1 Department of Pathology and Medical Biology, University of Groningen, University Medical Center Groningen, Hanzeplein 1 (EA11), 9713 GZ Groningen, the Netherlands; 2 W.J. Kolff Institute for Biomedical Engineering and Materials Science-FB41, University of Groningen, University Medical Center Groningen, Groningen, the Netherlands; 3 Groningen Research Institute for Asthma and COPD (GRIAC), University of Groningen, University Medical Center Groningen, Groningen, the Netherlands

**Keywords:** Comparative analysis, Preconditioning, Mesenchymal stromal cells, Adipose stromal cells, Proteomics

## Abstract

The secretome of adipose-derived stromal cells (ASC) is a heterogeneous mixture of components with a beneficial influence on cellular microenvironments. As such, it represents a cell-free alternative in regenerative medicine therapies. Pathophysiological conditions increase the therapeutic capacity of ASC and, with this, the benefits of the secretome. Such conditions can be partially mimicked *in vitro* by adjusting culturing conditions. Secretomics, the unbiased analysis of a cell secretome by mass spectrometry, is a powerful tool to describe the composition of ASC secretomes. In this proteomics databases review, we compared ASC secretomic studies to retrieve persistently reported proteins resulting from the most explored types of culturing conditions used in research, i.e., exposure to normoxia, hypoxia, or cytokines. Our comparisons identified only eight common proteins within ASC normoxic secretomes, no commonalities within hypoxic ASC secretomes, and only nine within secretomes of ASC exposed to proinflammatory cytokines. Within these, and regardless of the culturing condition that stimulated secretion, a consistent presence of extracellular matrix-related pathways associated with such proteins was identified. Confounders such as donors' age, sex, body mass index, the anatomical area where ASC were harvested, secretome collection method, data description, and how the data is shared with the scientific community are discussed as factors that might explain our outcomes. We conclude that standardization is imperative as the currently available ASC secretomic studies do not facilitate solid conclusions on the therapeutic value of different ASC secretomes.

**Graphical abstract:**

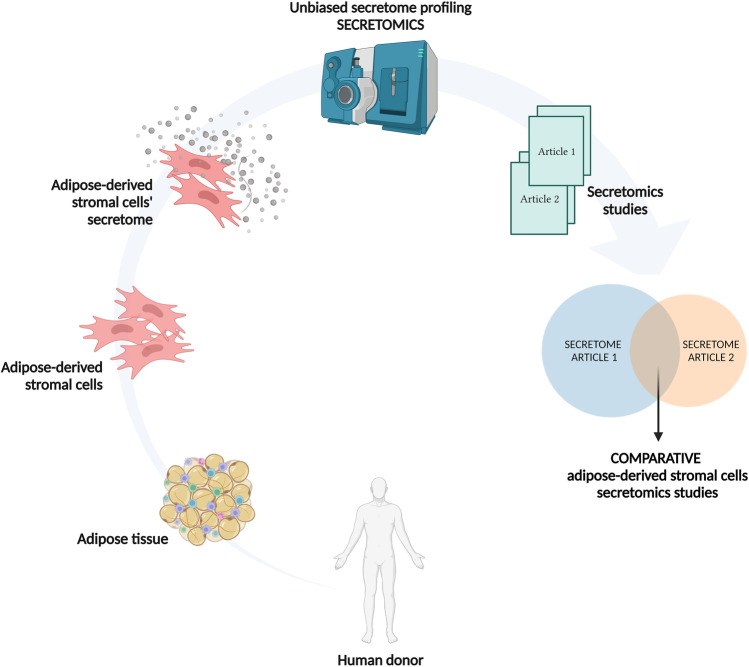

**Supplementary Information:**

The online version contains supplementary material available at 10.1007/s12015-023-10567-5.

## Introduction

Adipose tissue-derived stromal cells (ASC) are mesenchymal stromal cells (MSC) isolated and cultured from different adipose tissue (AT) depots. These cells have great potential for regenerative medicine, tissue engineering, and stem cell therapy. Their therapeutic potential is mainly based on their ability to stimulate tissue repair and remodeling [1–3]. They do this by stimulating processes of angiogenesis [3], mitogenesis [4], cellular migration [5], and suppressing apoptosis [6]. Furthermore, ASC also have immunomodulatory activities harnessed in the treatment of degenerative, inflammatory, and autoimmune diseases [7–9]. The ASC paracrine factors, i.e., the ASC secretome, play an important role in all these processes. Examples of the key factors in the ASC secretome are vascular endothelial growth factor (VEGF), platelet-derived growth factor (PDGF), epidermal growth factor (EGF), hepatocyte growth factor (HGF), insulin-like growth factor (IGF), fibroblast growth factor (FGF), monocyte chemoattractant protein-1 (MCP-1), various interleukins (ILs), prostaglandin E2 (PGE2), and others. Therefore, the ASC secretome is suggested to be instrumental in providing many of the ASC therapeutic effects [10].

The ASC secretome, as a cell-free product, is seen as a safer therapeutic strategy over ASC cell-based approaches. It eliminates risks of potential complications associated with the use of intact cells such as immune rejection, tumorigenesis, or cellular overgrowth, mitigating potential ethical concerns [10, 11]. In addition, secretomes can be produced in bulk, stored safely for longer periods of time, and transported more easily. Besides the cost-effective benefits, secretome production and quality control can be more easily standardized, which ensures consistent product quality and efficacy. To predict the therapeutic value of the ASC secretome, secretomics is a tool that allows us to unravel the secretome composition and discover unknown players [12–21]. *In vitro,* the secretome of ASC alters as the cells adapt to changes in their microenvironment, such as hypoxia or inflammatory stimuli [20, 21]. Different culturing conditions replicate physiological conditions that occur *in vivo* as part of tissue repair and regeneration and are the rationale for enhancing the ASC therapeutic capacity.

In this proteomics review, we used curated online databases and published literature to retrieve persistently reported proteins in ASC secretomes from commonly used culturing conditions. We discuss the most important confounders – donors' age, sex, body mass index, anatomical area of ASC harvesting, data description, and data sharing – that should be considered when moving towards standardization of human ASC secretomes for therapeutic application. Moreover, we performed further analysis using bioinformatic tools to seek culturing conditions-specific common proteins. This allowed us to identify extracellular matrix (ECM)-associated pathways as the most often associated biological network in the ASC secretome.

## Comparison criteria

Data were extracted from nine ASC secretomic studies [12–20] (Supplementary Table S1). Cell passage number (Supplementary Table S1; ranging from p1 to p35) and confluence (usually 80 to 100%) of the cells varied among the studies. All studies used human ASC cultured in different types of serum-free basal media for the secretome collection. The described secretomes were a result of ASC culturing exposed to normoxia (standard culture; 21 % oxygen) [11–19], hypoxia (1 or 5 % oxygen) [17, 20], or proinflammatory cytokines such as the tumor necrosis factor-α (TNF-α) and IL-1β [15, 16] (Tables 1 and 2, and tables S1-S6 in the Supplementary Material). A list of identified proteins in a format that allowed re-analysis was available for all studies. For the comparisons, the studies were clustered according to the explored (pre)conditioning methods and exposure times (Tables 1 and 2). As the protein names were used as the variables for the comparisons, all lists of secretomes were manually curated to ensure consistency in naming to minimize potential biases that could hinder our comparisons. All protein names were manually harmonized across all the lists – abbreviations were converted into the protein's full name; misspellings were rectified; duplicated proteins within a list were deleted (Supplementary Table S1). When not correctly described by the authors, protein isoforms and subunits could not be distinguished - which biased our comparisons. The contents of the final lists were compared using an online tool that identified the overlapping elements (https://bioinformatics.psb.ugent.be/webtools/Venn/). The common proteins found within each cluster were submitted to the PANTHER database for an overview of the functional biological processes and molecular function enrichment (Supplementary Table S7).

**Table 1:** Studies describing the ASC secretome resultant from ASC cultured under normal culturing conditions.

Strategy, duration	Author, year	Secretome profiling technique	Proteins identified	Commonly described proteins	
Normoxia, 16 hours	Zvonic *et al.*, 2007 [12]	2D-gel, Q-TOF MS	129	5	Ferritin (heavy chain); Ferritin (light chain); Quinone oxidoreductase; Dermatopontin; Xaa-Pro dipeptidase.
	Kehl *et al.*, 2019 [13]	LC-MS/MS	685		
Normoxia, 48 hours	Chiellini *et al.*, 2008 [14]	1D-gel, LC-MS/MS	73	9	Collagen type I, alpha 1; Collagen, type I, alpha 2; Collagen, type III, alpha 1; Collagen type VI alpha 1; Collagen type VI alpha 2; Lumican; Insulin-like growth factor binding protein 7; Fibronectin 1; Osteonectin.
	Lee M. J. *et al.*, 2010 [15]	LC-MS/MS	187		
	Kalinina *et al.*, 2015 [17]	Nano LC-MS/MS	100		
	Nakashima *et al.*, 2018 [18]	Nano LC-MS/MS	109		
	Lee Y *et al.*, 2021 [16]	LC-MS/MS	272		
Normoxia, 72 hours	Amodeo *et al.*, 2019 [19]	Nano LC-MS/MS	357	24	Albumin; Collagen, type III, alpha 1; Fibulin-1; Collagen, type I, alpha 1; Complement component 3; Pentraxin-related proteins PTX3; Collagen, type VI, alpha 1; Beta-mannosidase; Collagen, type I, alpha 2; Insulin-like growth factor binding protein 7; Fibronectin 1; Transforming growth factor-beta-induced protein ig-h3; Apolipoprotein A-1; Gelsolin; Cystatin-C; Decorin; Insulin-like growth factor binding protein 3; Lumican; Vimentin; Alpha-2-HS-glycoprotein; Alpha-fetoprotein; Lactotransferrin; Golgi membrane protein 1; Osteonectin.
	Frazier *et al.*, 2013 [20]	2D-LC-MS/MS	71

**Table 2:** Studies describing the ASC secretome resultant from ASC cultured under hypoxia and exposure to proinflammatory cytokines culturing conditions.

Strategy, duration	Author, year	Secretome profiling technique	Differentially secreted (vs. each study control – normal culturing condition)	
Hypoxia, 48 hours	Kalinina *et al.*, 2015 [17]	Nano LC-MS/MS	14	ADAMTS-like 1, inter-alpha (globulin) inhibitor H2, EGF-like repeats and discoidin I-like domains 3, ribonuclease RNase A family 4, adrenomedullin, tyrosine-protein phosphatase non-receptor type substract 1, fibrillin 2, glypican 1, transketolase, cartilage associated protein, heat shock protein 7- kDa family member 13, reticulocalbin 2, peroxiredoxin 3, proteasome activator subunit 2.
Hypoxia, 72 hours	Frazier *et al.*, 2013 [20]	2D-LC-MS/MS	5	Fibronectin-1, Transforming growth factor-beta-induced protein ig-h3, osteonectin, and collagens type I alpha 1, and alpha 2.
Cytokines – TNF-α, 48 hours	Lee M. J., 2010 [15]	LC-MS/MS	118	Numerous inflammatory cytokines and chemokines such as IL-6, IL-8, MIP-2R/CXCL2, CXCL5, CXCL6/GCP-2, CXCL10, and MCP-1/CCL2.
Cytokines – TNF-α, IL-1β, 48 hours	Lee Y *et al.*, 2021 [16]	LC-MS/MS	76	Numerous chemokines and extracellular matrix proteins such as CXCL6, CXCL2, CDCL3, collagen type I alpha 1, laminin subunitgamma-1, and lumican.

## Human ASC secretome after normoxic culturing

**Fig. 1:**
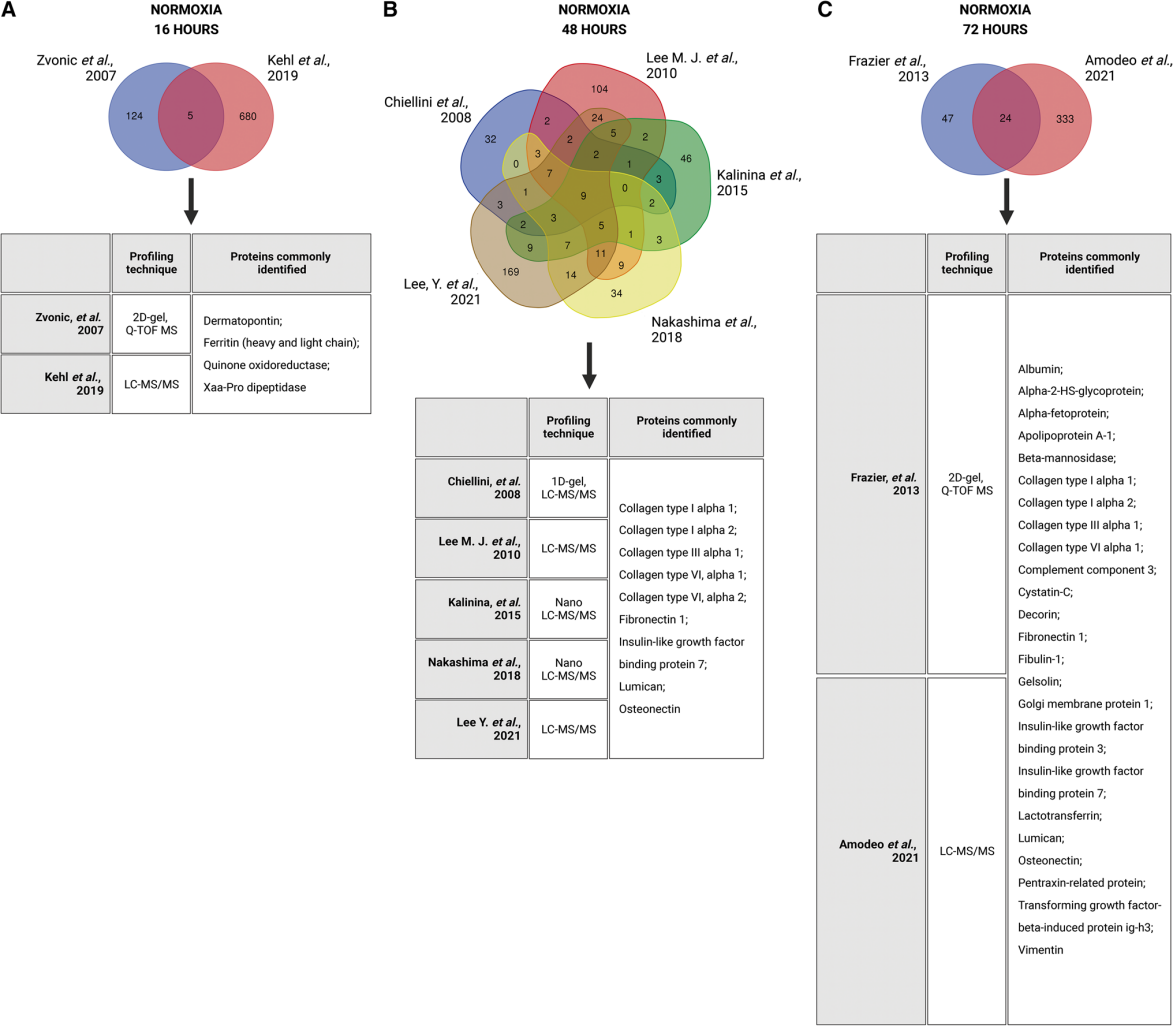
Comparative analysis of adipose-derived stem cells (ASC) secretomic studies clustered by normoxia preconditioning duration. Studies investigating normoxia preconditioning upon 16 (**A**), 48 (**B**), and 72 hours (**C**) are compared, and commonly secreted proteins are identified.

For our comparison, the ASC normoxic secretomes from these nine studies were grouped accordingly: 16 h [12, 13], 48 h [14–18], and 72 h [19, 20] exposure to normoxia (Table 1; Fig. 1). Each study had its unique set of identified proteins (Supplementary Tables S2-S4). The two studies composing the first cluster (16 h of normoxic culturing) [12, 13] reported, together, a total of 814 proteins (129 proteins [12], 685 proteins [13]), of which only five (0.61%) overlapped (Fig. 1A): dermatopontin, ferritin (heavy and light chain), quinone oxidoreductase, and Xaa-Pro dipeptidase. The five studies composing the second cluster (48 h of normoxic culturing) [14–18] reported in total, 740 proteins (72 proteins [14], 109 proteins [18], 187 proteins [15], 100 proteins [17], and 272 proteins [16]), and we identified only nine overlapping proteins (1.22%) (Fig. 1B): various collagen (COL) types, fibronectin 1 (FN1), IGF binding protein 7 (IGFBP-7), lumican, and osteonectin. The third and last cluster (72 h of normoxic culturing) reported, in total, 428 proteins (71 proteins (27), 357 (26) with 24 proteins in common (5.61%): albumin, alpha-2-HS-glycoprotein, alpha-fetoprotein, beta-mannosidase, various COL types, complement component 3, cystatin-C, decorin, FN1, gelsolin, Golgi membrane protein 1, IGFBP-3, lactotransferrin, lumican, osteonectin, pentraxin-related protein (PTX3), transforming growth factor (TGF) beta-induced protein ig-h3, and vimentin (Fig. 1C). This cluster represents the one with the highest percentage of similarities. Our analyses showed significant variability in secretome composition even during what is considered 'standard' ASC culture conditions.

**Fig. 2:**
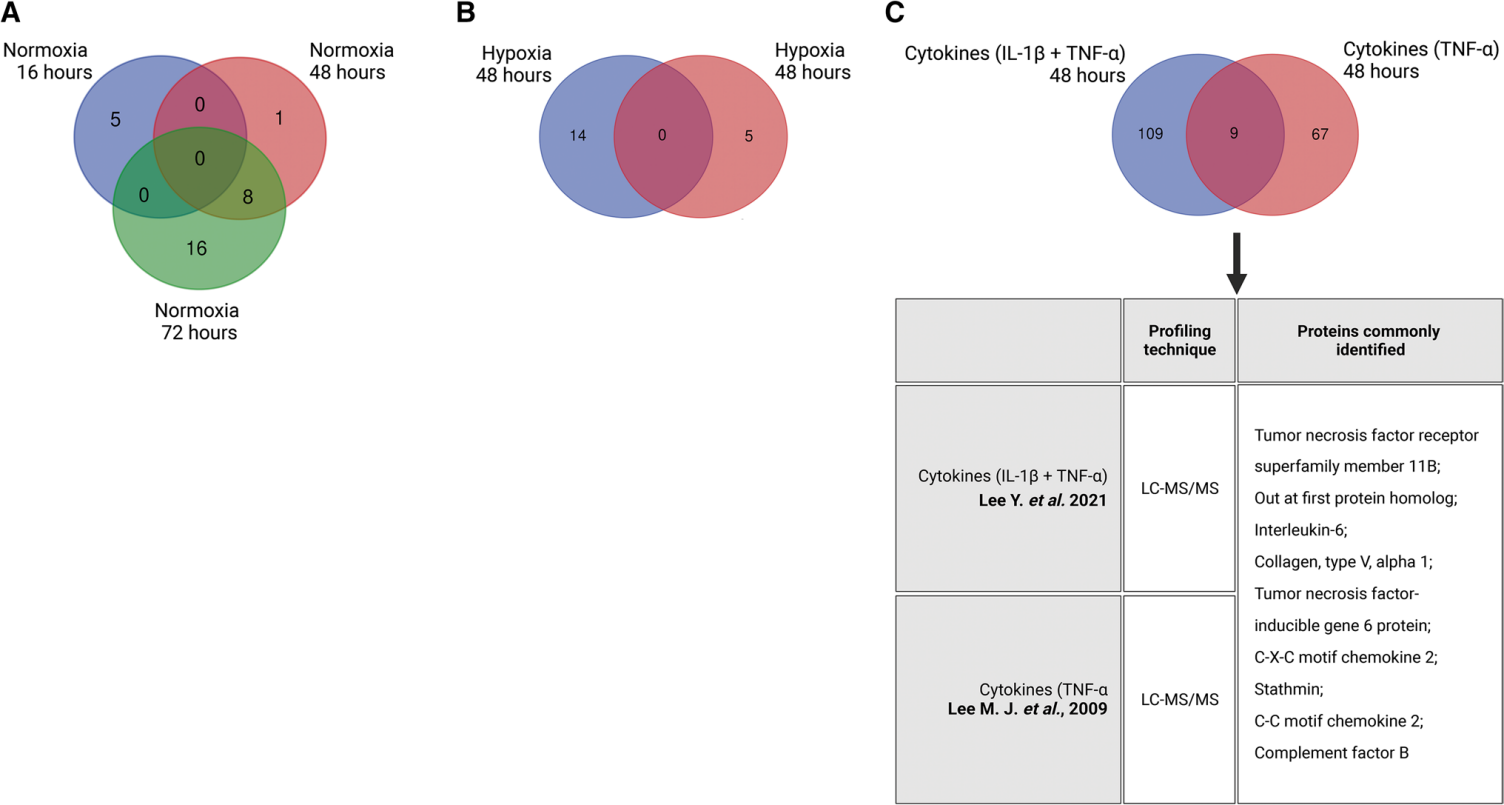
Comparative analysis among the core proteins present in the adipose-derived stromal cells (ASC) secretomes within each cluster. No proteins were identified among normoxic studies of all three culture durations, but eight common proteins were identified between studies describing the ASC secretome of normoxic culturing upon 48 and 72 hours (**A**); no common proteins were identified among the ASC hypoxia-dependent secretomes (**B**); and nine proteins were identified in the cytokines-dependent ASC secretomics studies (**C**).

Interestingly, no commonly secreted proteins could be identified when all the nine normoxic studies from all three time-points were compared among each other (Fig. 2A). However, eight common proteins could be found between the 48 and 72 h clusters only: collagen type I alpha 1 (COL1A1) and COL1A2, COL3A1, COL6A1 and COL6A2, FN1, lumican, and osteonectin (Fig. 2A). With these, a functional analysis was performed (Supplementary Table S7). Within the various processes of which these proteins are a part, ECM-associated processes were consistently detected. Gene ontology terms, such as ECM organization, cell-matrix adhesion, collagen biosynthetic and catabolic processes, and cellular response to TGF-β stimulus, were repeatedly reported by PANTHER.

## Human ASC secretome alterations by hypoxic culturing

Alternative strategies to standard culturing can induce changes in the ASC secretome due to adaptation to the environment, and various molecules may disappear or become more or less present. For instance, the production of various growth factors, such as VEGF, FGF, PDGF, HGF, or IGF, are reported to be increased by hypoxia [20, 22, 23]. Therefore, alternative culturing conditions are thus used to improve the secretome to better fit the therapeutic needs.

Table 2 and Supplementary Table S5 summarize the two available ASC hypoxic secretomic studies [17, 20]. The studies' methodology differs; the secretomes were collected after cells response to 48 h [17] or 72 h [20] and applied oxygenation of 1% [17] or 5% O_2_ [20]. Given these differences, we did not carry out clustering, and the studies were discussed separately. Both reported a small impact of hypoxia on the ASC secretome. In the first study, out of the 100 proteins found upon normoxia, 14 (14%) were differently secreted upon hypoxia [17] (Table 2) – six proteins that were present were not found in the normoxic secretomes, and 8 were found in the hypoxic samples less frequently compared to normoxia. In the second study, out of the 71 proteins found in the normoxic ASC secretome, five were reported as differentially secreted (7%) upon hypoxic culturing [20]. Although not clustered according to our criteria, we have compared the proteins identified by both studies, detected no similarities, and therefore did not proceed with functional analyses (Fig. 2B). Interestingly, both studies reported that hypoxic culturing reduces the presence of ECM-related proteins in the ASC secretome.

## Human ASC secretome following exposure to cytokines

*In vitro*, the secretory properties of ASC can also be altered by exposure to proinflammatory cytokines [15, 16]. Within the nine studies we selected, two described ASC secretome changes upon exposure to proinflammatory cytokines for 48 h (Table 2, Supplementary Table S6). The first reported changes in the secretion of ASC exposed to TNF-α [15]. Out of the 187 proteins reported in the non-stimulated (normoxic) ASC secretome (Table 1), 118 (63.1%) were differentially secreted by TNF-α-stimulated ASC (Table 2). These differentially secreted proteins included cytokines, chemokines, and inflammatory mediators (i.e., IL-8, C-C motif chemokine 2 (CCL2), C-X-X motif chemokine 6 (CXCL2), complement C1S, and C1r subcomponent), as well as ECM-associated proteins (i.e., matrix metalloproteinase 1 and PTX3). The second study reported changes in the ASC secretome upon ASC culturing with a cocktail of TNF-α and IL-1β [16]. 272 proteins were described in the non-stimulated (normoxic) ASC secretome (Table 1), and 76 (27.9%) were differentially secreted upon proinflammatory stimulation (Table 2). Within the differentially secreted proteins, ECM components (various collagen types, vimentin, and gelsolin), growth factor-associated molecules such as different IGFBPs, and inflammation-associated factors such as different cytokines and chemokines (i.e., C-C motif chemokines, cytokine receptor-like factor 1, IL-11, IL-6, TNF-inducible gene 6 protein (TSG-6), and TNF receptor superfamily member 11B (TNFRSF11B) [16].

Both studies, together, reported 194 proteins differentially secreted upon cytokine exposure. With these, we performed our comparative analysis (Fig. 2C). Nine proteins were commonly present: TNFRSF11B, out at first protein homolog (OAF), IL-6, COL5A1, TSG-6, CXCL2, stathmin, CCL2, and complement factor B (CFB). Submitted to functional analysis, most of these proteins (except the OAF protein) revealed involvement in ECM-associated processes (Supplementary Table S7). It might seem unexpected that proteins secreted by ASC exposed to cytokines are involved in ECM pathways, but inflammation-associated proteins, such as TSG-6, IL-6, TNFRS11B, CXCL2, CCL2, and CFB, have already been associated with various ECM components and processes. TSG6 interacts with the cluster of differentiation 44 (CD44) [24]; therefore, it is implicated in ECM key signaling pathways. IL-6 is strongly associated with ECM remodeling and can induce different ECM profiles [25]. TNFRS11B is a circulating glycoprotein involved in the development of fibrosis and the progression of several human malignancies [26]. CXCL2 is involved in cell adhesion to ECM proteins via the CXCL2-CXCR2 signaling axis [27], and CCL2 is involved in the ECM turnover via CCL2/MCP-1 signaling [28].

## Confounders hindering ASC secretomics studies comparisons

### Confounders playing a role prior to the ASC preconditioning

The microenvironment (physical, chemical, and biological factors present both *in vivo* and *in vitro*) can dictate phenotypical and functional changes in ASC and subsequently modulate the secretome. Although the International Society for Cellular Therapy (ISCT) has defined the following criteria for MSC: plastic adherent, capable of differentiating into multiple lineages, positive for the surface markers CD105, CD90, CD73, and negative for CD45, CD34, CD11b, CD19, and HLA-DR [29]; not all studies report about their characterization or use the same criteria. Therefore, this confounder will affect ASC secretomic studies. Variations in phenotypes (and morphology) have been extensively discussed by Tan, L. *et al.* [30] and will not be further discussed here. Donor variation is another important factor (i.e., donor age, sex, BMI, physical condition, presence of underlying (metabolic) disease, and harvesting area of AT) that influences the phenotype of cultured ASC [25–27], often in an unpredictable fashion, which directly impacts the secretome.

#### Age

Donor age, as well as aging through prolonged cell passaging, causes senescence, increased apoptosis, and reduced proliferation and differentiation capacity and, therefore, negatively impacts the phenotype of ASC [31–33]. In contrast, other studies show that donor age does not impact proliferation and differentiation [34, 35]. The impact of donor age and prolonged passaging on secretome composition has not yet been investigated. The studies we analyzed used either pooled samples of cultured ASC from donors aged under 50 [14, 17, 18, 20], under 90 [13], or did not disclose the donors' age [12, 15, 16, 19].

#### Sex

While ASC are generally cultured from lipoaspirates retrieved from females, most studies are biased toward the female sex. ASC from females consistently suppressed immune responses *in vitro* more than ASC from male donors [36]. This was attributed to higher concentrations of anti-inflammatory mediators in the secretome, such as indoleamine 2,3-dioxygenase 1, IL-1 receptor antagonist protein, and PGE_2_ [36]. The female sex also augments the proliferation and differentiation of ASC [37]. Most studies selected for our analysis used ASC from female donors to generate secretomes [12, 13, 17, 18, 20]. Others pooled male and female donors [19], and yet others did not disclose this information [15, 16]. An interesting – indirect – finding regarding donors' sex and ASC secretome composition was reported by Kalinina *et al*. [17]. Their study reported no more than 1/6th of overlap (100 proteins out of 606) among normoxic ASC secretomes collected from 10 different ASC cultures – cells harvested from 10 female donors under 50. This shows that even within a study that uses donors of the same sex and similar age, inter-donor variation plays a decisive role in the ASC secretome composition. Further standardized research is needed to confirm the potential advantages of donor matching for ASC usage, especially regarding the impacts on the secretome.

#### Body mass index

Donors with obesity display an excess in adipocyte generation, which results in ASC with altered immunophenotypes, increased capacity to differentiate into adipocytes, reduced proliferation, migration, viability, and altered inflammatory transcriptome [38, 39]. Senescence-associated characteristics were also reported in ASC from obese donors [40]. Global changes in the ASC secretome have never been investigated in this context. However, inflammatory proteins such as IL-1β, IL-8, and MCP-1, for example, were upregulated in ASC obtained from subjects with obesity [41]. This modulation is associated with the development of low-grade chronic systemic inflammation characteristic of obesity. As for the studies investigated for this review, information regarding BMI was mostly absent.

#### Anatomical area

Considering the heterogeneity of MSC function, proliferation, and differentiation potential among tissues (e.g., adipose versus bone marrow) [37, 42] and the existence of heterogeneous ASC populations in a single location [17, 43, 44], it is likely that ASC from different AT depots have different therapeutic potential. Differences in the expression profiles of developmental genes were shown between ASC sourced from different depots [45]. An intertwined influence of gender and the anatomical area has also been proposed [46, 47]. However, the global composition of ASC secretomes from different anatomic areas has not yet been investigated. Most studies presented here used subcutaneous ASC for the secretomes description [12, 13, 16, 17, 19, 20]. One explored the secretome of gonadal-derived AT [14], and others did not disclose the ASC source [15, 18].

## Confounders playing a role during the secretome collection method, protein detection, and data analysis

Studies of the cell secretome have significantly increased over the years. The development of high-precision proteomic platforms and mass spectrometry (MS) instruments are the main factors enabling the generation of high-quality data and in-depth descriptions of various secretomes. All the studies included in the present review used MS for protein detection, varying the separation technique and the performance of additional assays for validation (Supplementary Material). Some studies have separated proteins in one dimension (1D) or 2D gels to explore them further using MS [12, 14, 20]. Others have used liquid chromatography (LC) [13, 15, 16], 2-dimensional (2D) LC [20], or Nano LC [17–19]. To date, nano-LC-MS is the preferred technique used in secretomics because of its higher sensitivity compared to the other commonly used MS strategies [48].

The secretome sample quality is as important as the MS instrument for protein detection. Reduced levels or preferably lack of serum in the sample and a sufficiently high protein concentration are crucial for optimal detection. However, the reduced presence or absence of serum-associated growth factors during ASC culturing will affect the secretomic results. This means that the identified proteins are not only a reflection of the culturing condition but also of the serum deprivation duration. Serum deprivation effects are often neglected but must be taken into consideration as it has been shown to induce reactive oxygen species production [49], cell autophagy and apoptosis [50], and changes in cellular phenotype [51]. Furthermore, ASC are usually cultured and preconditioned in a 2D environment. Upon secretome collection, the sample is concentrated by ultrafiltration through filters made of various molecular weight cut-offs – a technique used by all the studies presented here.

*In silico* methods that rely on database annotations or protein sequence information are mainly used for secretome analysis. The main challenge here is to render the identified datasets available in an analyzable fashion for the scientific community. Our ASC secretome comparative analysis was limited because the lists of proteins lacked appropriate (protein) identifiers or adequate protein nomenclature. This resulted in a loss of recognition of bona fide-secreted proteins. We focused on extracting and comparing qualitative data because quantitative data were hardly available. Furthermore, analyses may also be limited due to challenges in the choice of parameters like cut-offs or handling missing data points. These factors significantly affect the composition of a described secretome.

## Reporting ASC secretomics studies outcomes

Many of the previously discussed confounders are difficult to circumvent. However, reporting all relevant experimental details in publications – cells’ isolation, cell characterization, secretomes collection, protein detection, databases and software used for data analysis – will facilitate the comparisons and replication by other researchers. Moreover, a possible calibrator that can sensibly improve secretomics studies is the standardization of how these studies' outcomes – protein lists – are interpreted and reported. If each study decides to generate a secretome catalog using its own conventions, global comparisons, such as the one we aimed to conduct, will always be hindered. Proteins identifiers such as the ones assigned by UniProt – UniProt entries – can easily be incorporated as a standard format for publicly available secretomics data. The use of publicly available repositories to store raw data is crucial, as well as robust, durable user-friendly file formats for consultation and re-analysis. This strategy will facilitate an appropriate description of the proteins identified within a study and support the re-analysis of such available data.

## Concluding remarks

The comparisons of ASC secretome studies revealed the difficulties of identifying common secreted proteins within the subsets available in the literature. ECM-associated proteins, including various collagen types, proteoglycans, and glycoproteins, were persistently detected regardless of the preconditioning and culturing exposure time considered. Factors contributing to variation in ASC secretome data from secretomics studies are donors' intrinsic characteristics, ASC culturing and preconditioning strategies, and the final format to describe the secretome composition results. These factors must be considered when standardizing the human ASC secretomes. Standardization is imperative as the current ASC studies do not facilitate solid conclusions regarding the therapeutic application. Standardization will optimize and unify studies, reducing literature inconsistencies, allowing solid interpretation of ASC secretomics investigations, and moving the field towards clinical application.

### Supplementary Information

ESM 1. The Supplementary Material is composed of seven tables (tabs S1-S7): S1. Detailed overview of the nine ASC secretomics studies analyzed. S2. List of proteins identified by ASC secretomics studies exploring 16 hours of normal culturing. S3. List of proteins identified by ASC secretomics studies exploring 48 hours of normal culturing. S4. List of proteins identified by ASC secretomics studies exploring 72 hours of normal culturing. S5. List of proteins identified by ASC secretomics studies exploring hypoxic culturing. S6. List of proteins identified by ASC secretomics studies exploring exposure to proinflammatory cytokines as a culturing condition. S7. Gene Ontology analysis of the common proteins found within ASC secretomes resulted from normal culturing (48 and 72 hours), and within ASC secretomes resulted of proinflammatory cytokines exposure - biological processes and molecular function enrichment. (XLSX 101 kb)

## Data Availability

Not applicable.
